# Consistent hypersocial behavior in mice carrying a deletion of *Gtf2i* but no evidence of hyposocial behavior with *Gtf2i* duplication: Implications for Williams–Beuren syndrome and autism spectrum disorder

**DOI:** 10.1002/brb3.895

**Published:** 2017-12-19

**Authors:** Loren A. Martin, Erica Iceberg, Gabriel Allaf

**Affiliations:** ^1^ Department of Graduate Psychology Azusa Pacific University Azusa CA USA; ^2^ Department of Biology and Chemistry Azusa Pacific University Azusa CA USA

**Keywords:** 7q11.23, autism, social motivation, TFII‐I, Williams syndrome

## Abstract

**Introduction:**

Williams–Beuren syndrome (WBS) is a developmental disorder caused by hemizygous deletion of human chromosome 7q11.23. Hypersocial behavior is one symptom of WBS and contrasts with hyposociality observed in autism spectrum disorder (ASD). Interestingly, duplications of 7q11.23 have been associated with ASD. The social phenotype of WBS has been linked to *GTF2I* or general transcription factor IIi (*TFII‐I*). Duplication of *GTF2I* has also been associated with ASD.

**Methods:**

We compared mice having either a deletion (*Gtf2i* ^*+/−*^) or duplication (*Gtf2i* ^*+/dup*^) of *Gtf2i* to wild‐type (*Gtf2i* ^*+/+*^) littermate controls in a series of behavioral tasks including open‐field activity monitoring, olfactory probes, a social choice task, social transmission of food preference, habituation–dishabituation, and operant social motivation paradigms.

**Results:**

In open‐field observations, *Gtf2i* ^*+/−*^ and *Gtf2i* ^*+/dup*^ mice demonstrated normal activity and thigmotaxis, and surprisingly, each strain showed a significant preference for a stimulus mouse that was not observed in *Gtf2i* ^*+/+*^ siblings. Both *Gtf2i* ^*+/−*^ and *Gtf2i* ^*+/dup*^ mice demonstrated normal olfaction in buried food probes, but the *Gtf2i* ^*+/−*^ mice spent significantly more time investigating urine scent versus water, which was not observed in the other strains. *Gtf2i* ^*+/−*^ mice also spent significantly more time in nose‐to‐nose contact compared to *Gtf2i* ^*+/+*^ siblings during the open‐field encounter of the social transmission of food preference task. In operant tasks of social motivation, *Gtf2i* ^*+/−*^ mice made significantly more presses for social rewards than *Gtf2i* ^*+/+*^ siblings, while there was no difference in presses for the *Gtf2i* ^*+/dup*^ mice.

**Discussion:**

Results were remarkably consistent across testing paradigms supporting a role for *GTF2i* in the hypersocial phenotype of WBS and more broadly in the regulation of social behavior. Support was not observed for the role of *GTF2i* in ASD.

## 1 INTRODUCTION

Williams–Beuren syndrome (WBS), or Williams syndrome, is a rare developmental disorder caused by a hemizygous deletion on human chromosome 7q11.23 typically spanning 1.55 Mb and encompassing 26 genes, although approximately 5% of cases are due to a larger 1.84‐Mb deletion including 28 genes (Bayes, Magano, Rivera, Flores, & Perez Jurado, 2003). It is reported to occur in as high as 1/7,500 births, but often remains undiagnosed (Stromme, Bjornstad, & Ramstad, 2002). The initial symptoms of WBS that were described included intellectual disability, abnormal facial features, supravalvular aortic stenosis, and growth retardation (Beuren, Apitz, & Harmjanz, 1962; Williams, Barratt‐Boyes, & Lowe, 1961). Further characteristics of dental anomalies, additional cardiovascular abnormalities, and a hypersocial and friendly personality were added to the phenotype after more research had been conducted (Mervis et al., 2000; Pober, 2010). The hypersocial phenotype has been further characterized by a proclivity for direct eye contact, attraction to social interactions with strangers, bias for focusing on faces and eyes, positive affect, and insensitivity for negative affect suggestive of decreased social anxiety (Bellugi, Adolphs, Cassady, & Chiles, 1999; Doyle, Bellugi, Korenberg, & Graham, 2004; Jarvinen, Korenberg, & Bellugi, 2013; Jarvinen‐Pasley et al., 2008). The hypersocial phenotype may also relate to increased language abilities of WBS children (Mervis & Robinson, 2000) as they have been shown to infuse their storytelling with more affective language and engage the audience more than those with Down syndrome (Bellugi, Korenberg, & Klima, 2001; Jones et al., 2000). Despite having numerous social interactions, individuals with WBS often have few friends (Frigerio et al., 2006).

Compared to WBS, autism spectrum disorder (ASD) is a much more widely known and diagnosed developmental disorder with an estimated prevalence rate of 1 in 68 in the United States (Christensen et al., 2016). It is characterized by a wide variety of phenotypes with varying levels of severity between individuals (Sasson, Nowlin, & Pinkham, 2013). Those with ASD demonstrate symptoms in two distinct categories: (i) “persistent deficits in social communication and social interaction” and (ii) “restricted, repetitive patterns of behavior, interests, or activities” (Association, 2013).

The social difficulties observed in ASD individuals have been postulated to stem from a lack of social motivation (Chevallier, Kohls, Troiani, Brodkin, & Schultz, 2012). The social motivation theory postulates that social motivation is a set of biological mechanisms that present social interactions as inherently rewarding and motivating for typically developing individuals (Chevallier et al., 2016). Those diagnosed with ASD have early deficits in social cognition that are thought to reduce social interest and motivation, leading to the theory that because those with ASD do not find social interactions rewarding, they are not motivated to seek them out (Novacek, Gooding, & Pflum, 2016). Several recent studies have found support for the social motivation theory including a preference of ASD individuals for nonsocial over social videos (Dubey, Ropar, & Hamilton, 2017), a reduced preference for social stimuli in toddlers diagnosed with ASD (Ruta et al., 2017), and an inverse relationship between ASD traits and self‐reported pleasure from social interactions (Novacek et al., 2016).

The genetic cause of Williams syndrome was first discovered in 1993 (Ewart et al., 1993). Typically, the severity of the disorder is caused by the specific genes within the 7q11.23 chromosomal region, known as the Williams syndrome critical region (WSCR), that have been deleted with more severe cases having larger deletions. Repeated genes flanking the WSCR become misaligned during meiosis and lead to nonallelic homologous recombination that can cause these microdeletions (Bayes et al., 2003).

Most symptoms of WBS have been shown to also occur with the heterozygous deletion of the WSCR in mice (Segura‐Puimedon et al., 2014). Clues toward the genetic basis of the hypersocial phenotype of WBS were provided through the study of separate mouse lines carrying partially overlapping half‐deletions of the WSCR. Mice carrying a proximal deletion exhibited increased social interest compared to controls, whereas mice carrying a distal deletion demonstrated normal social behavior coupled with cognitive impairments (Li et al., 2009). An additional study of a WBS patient with an atypical deletion suggested *GTF2I* as the gene behind the hypersocial phenotype (Dai et al., 2009). The development of a mouse line carrying a heterozygous deletion of *Gtf2i* provided support for a role of this gene in the social phenotype of WBS (Sakurai et al., 2011). Further support for this gene–behavior relationship was demonstrated in subsequent studies, including the rescue of the phenotype using gene therapy in *Gtf2i* KO mice (Borralleras, Sahun, Perez‐Jurado, & Campuzano, 2015), as well as the association of low social anxiety and social communication with *GTF2I* SNPs in the general population (Crespi & Hurd, 2014).

Williams–Beuren syndrome is a well‐defined disorder with a known genetic cause. In contrast, ASD is more loosely defined with mostly unknown etiology, although there is evidence for strong genetic contributions including several known genetic risk factors (Shailesh, Gupta, Sif, & Ouhtit, 2016). While the deletion of the 7q11.23 chromosomal region results in the Williams syndrome phenotype, duplication of this critical region results in an opposite phenotype similar to ASD in regard to language abilities (i.e., speech delays), visual–spatial processing, and behavior such as decreased social interaction, functionally impairing anxiety, and repetitive behavior (Berg et al., 2007; Depienne et al., 2007; Malenfant et al., 2012; Van der Aa et al., 2009). The observation of autistic behaviors in individuals with this duplication suggests that there is a gene in the WSCR, such as *GTF2I*, that contributes to the ASD phenotype. Indeed, *GTF2I* SNPs have been linked to ASD (Malenfant et al., 2012), and duplication of this gene in both humans and mice has been associated with increased separation anxiety (Mervis et al., 2012). However, the social behavior of mice carrying a *Gtf2i* duplication has not been thoroughly explored.

The *General Transcription Factor 2i* (*GTF2I*) gene, found in both humans and mice, encodes for the GTF2I protein. It functions in the regulation of transcription by interacting with tissue‐specific transcription factors. GTF2I has also been identified as a downstream target in various signal transduction cascades (Sacristan et al., 2009). The objective of our study was to thoroughly explore the role of the *Gtf2i* gene in the hypersocial behavioral phenotype of WBS and the hyposociality of ASD, with a broader emphasis on its role in the regulation of social behavior. We compared mice with a heterozygous deletion of the *Gtf2i* gene (*Gtf2i* ^*+/−*^) and a duplication of the *Gtf2i* gene (*Gtf2i* ^*+/dup*^) with wild‐type (*Gtf2i* ^*+/+*^) sibling controls in a series of paradigms aimed at further characterizing and confirming the relationship of this gene to social behavior.

## 2 METHODS AND MATERIALS

### 2.1 Subjects

*Gtf2i* ^+/−^ × *Gtf2i* ^+/+^ and *Gtf2i* ^*+/dup*^ × *Gtf2i* ^+/+^ breeder pairs were obtained from the laboratory of Lucy Osborne at the University of Toronto and had been previously backcrossed onto the C57BL/6 background strain. The production of these mice is described in Mervis et al., 2012 (Mervis et al., 2012). Male *Gtf2i* ^+/−^ and *Gtf2i* ^*+/dup*^ mice were mated with female *Gtf2i* ^+/+^ (wild‐type) mice to yield litters that were approximately 50% each genotype. The male offspring were then used as test subjects in the behavioral experiments. Genotyping was conducted by Transnetyx (Memphis, TN) using real‐time PCR assays developed from sequencing data. This genotyping was confirmed in‐house by the PI using PCR protocols and primers generously provided by Dr. Osborne and previously published by Mervis et al. (2012).

All mice were greater than 8 weeks of age when testing commenced and completed testing before 1 year of age. Also, the same subjects (15 *Gtf2i* ^*+/−*^ mice and 14 *Gtf2i* ^*+/+*^ siblings; 10 *Gtf2i* ^*+/dup*^ mice and 14 *Gtf2i* ^*+/+*^ siblings) were used in all of the experiments with the exception of the habituation–dishabituation paradigm in which a separate group of eight *Gtf2i* ^*+/−*^ mice and eight *Gtf2i* ^*+/+*^ mice were tested following the completion of all other tests. Otherwise, repeated testing of the same subjects was conducted in the order listed below. Sample size was smaller in the social transmission of food preference task due to the lack of adequate food consumption by some of the assigned demonstrator mice. One *Gtf2i* ^*+/−*^ mouse did not complete the operant paradigms due to health issues. All experimenters conducting tests were blind to the genotype. Male C57BL/6J mice were used as stimulus partners for the test mice and were age‐matched with the exception of the habituation–dishabituation paradigm in which juvenile stimulus partners were utilized. C57BL/6J mice were obtained from the principal investigator's own breeding colonies that were originally established from breeder pairs obtained from Jackson Laboratories (Bar Harbor, ME, USA).

All mice were housed in a vivarium with a set 14:10‐hr light:dark cycle in a climate‐controlled setting with temperature maintained at 20°C. All testing was conducted during the light phase of the cycle. Mice were housed in groups of 2–4 in ventilated cages (OptiMICE; Animal Care Systems, Centennial, CO, USA) and given pellet feed (Purina 5001) and water ad libitum. Mice were identified via tail tattoos. All procedures were approved by the Azusa Pacific University Institutional Animal Care and Use Committee, and all mice were treated in accordance with the NIH guidelines for the care and use of animals in research.

### 2.2 Equipment

#### 2.2.1 Open‐field tests

A Single Unit Open‐Field Enclosure (San Diego Instruments, San Diego, CA, USA) was used to measure activity and to conduct the social choice task. Two halogen desk lamps with 35‐W bulbs were placed on opposite sides of the enclosure, 52 cm above the base of the arena floor, and angled so that they were directed toward the middle of the arena wall opposite each lamp. For the social choice paradigm, 2 black wire mesh pencil cups (10.5 cm base diameter and 13.5 cm tall) were placed in opposing corners of the enclosure and masked the movement of any stimulus mice placed under them. A camera (Model TG3Z2910AFCS, Computer Optics Group, Commack, NY, USA) was placed centrally 78 cm over the enclosure and connected to a laptop running the ANY‐maze video‐tracking system (San Diego Instruments). Virtual zones were created within the enclosure using the software program including a square center zone measuring 43 × 43 cm, a perimeter zone 7 cm from the enclosure walls, and for the social choice paradigm, perimeter zones 7 cm around each pencil cup.

#### 2.2.2 Operant tests

For the social motivation and valence comparison operant paradigms, we utilized four‐center channel modular shuttle boxes from Med Associates Inc. (model ENV‐010MC; St. Albans, VT, USA). Each box was divided into two chambers (the test chamber and target chamber) using an auto‐guillotine door (model ENV‐010B) covered by a wire grid that prevented mice from freely moving between chambers while also allowing social interaction between mice. Mice levers (ENV‐3010M; Med Associates) were placed opposite to this door in the right chamber (the test chamber) and were programmed to either open the door or deliver a food reward, depending upon the testing paradigm. A food reward consisting of 0.02 ml of a 2% sucrose and evaporated milk solution was dispensed via a liquid dipper (ENV‐202M‐S; Med Associates), which was located between the two mice levers. Each shuttle box was enclosed within a melamine sound‐attenuating cubicle (model ENV‐016MD; Med Associates). The operant programs were run using Med PC‐IV software from Med Associates using customized programs written in the laboratory.

### 2.3 Open‐field tests

Open‐field tests were conducted to measure any differences between the mice in activity, thigmotaxis, and social investigation. Mice were tested in three different 10‐min stages over two consecutive days with a 22‐ to 26‐hr difference in time between days. During the first day, the mice were allowed to run around freely for 10 min in the enclosure to acclimate to the arena. This helped reduce the effect of anxiety to a novel environment during the actual test days. Movements of the test mice were tracked during this time to determine total distance traveled as well as time spent within the perimeter and center zones. The C57BL6 stimulus mice were also acclimated to being placed under the pencil cups for 10 min.

On day two, the test mice and stimulus mice were, once again, acclimated for 10 min in the arena before being tested in the social choice paradigm (Moy et al., 2004). After acclimation, the test mice were put in the center of the arena and allowed to choose between a stimulus mouse in one corner of the arena (under a pencil cup) and an empty pencil cup in the opposite corner. The movements of the test mice were again tracked during the 10‐min test. The amount of time spent within the empty cup and stimulus mouse zones was recorded, as well as the total distance traveled.

### 2.4 Olfactory probes

#### 2.4.1 Buried food task

The buried food task (Yang & Crawley, 2009) was used to determine whether differences in olfactory ability may underlie differences in social behavior. Test mice were first deprived of mouse chow for 12–15 hr prior to testing. The test mice were then placed on the narrow end of a clean cage and recorded with ANY‐maze tracking software for 5 min or until they found a single Cocoa Puff (General Mills) located in the center of the of the cage 10 cm from the wide end and buried 1 cm below Sani‐Chips bedding (PJ Murphy, Montville, NJ, USA). The length of time it took each mouse to find the Cocoa Puff was recorded. Mice that did not find the buried food within 5 min were removed from the analysis. The clean cages were identical to the home cages of the mice in an effort to lower anxiety associated with a novel environment.

#### 2.4.2 Urine scent task

The urine scent task was designed to test the interest of each mouse to a social scent. Test mice were recorded for 10 min when simultaneously presented with two different slides in a clean cage. The first slide was painted with female mouse urine and clipped to one corner of the wide end of the cage with a binder clip. The second slide was painted with deionized water and clipped to the other corner of the wide end of the cage. Test mice were then placed on the opposite narrow end of the cage from the slides, and movements were tracked within the cage using ANY‐maze. Time spent on investigating the urine and waterslides was both recorded.

### 2.5 Social transmission of food preference

The social transmission of food preference was designed to see whether preference for a specific flavor transferred from one mouse to another via social interaction (Wrenn, Harris, Saavedra, & Crawley, 2003). The ANY‐maze video‐tracking software was used for this round of testing. Test and demonstrator mice were placed in individual cages with access to pure powdered mouse chow for 6 hr in order to become accustomed to eating powdered chow instead of pellets. All foods were then removed for 16–18 hr prior to testing. On the test day, demonstrator mice were given access to either 1% cinnamon or 2% cacao powdered chow for 1 hr. In order to be used as a demonstrator mouse, each mouse was required to eat at least 0.2 g of food. After the hour with the flavored chow, the demonstrator mice were placed in the home cage of the test mouse and allowed to interact for 30 min. The interactions between the mice were recorded using the ANY‐maze software. The number and total duration of nose‐to‐nose interactions between the test mouse and the demonstrator mouse were recorded. The demonstrator mouse was then removed from the cage after 30 min, and the test mouse was given access to weighed jars of 1% cinnamon and 2% cacao for 1 hr in their individual cage. The food jars were weighed before and after to determine food preference.

### 2.6 Habituation–dishabituation paradigm

The habituation–dishabituation paradigm was also conducted to determine whether the *Gtf2i* ^*+/−*^ mice demonstrated the expected pattern of habituation to a juvenile stimulus partner with repeated exposures followed by dishabituation when the stimulus partner was replaced with a novel juvenile mouse (Dantzer, Bluthe, Koob, & Le Moal, 1987; Winslow & Camacho, 1995). Fifteen minutes prior to conducting the paradigm, both the stimulus and test mice were separated into individual cages identical to their home cages. Once the test began, the juvenile stimulus mouse was placed in the center of the narrow end of the home cage of the test mouse. The social interactions between the two mice were observed for the duration of one minute, and the amount of time the test mouse spent on investigating its social partner was recorded. Such social investigations were defined as when the test mouse had its head directed toward and within 1 cm of the juvenile mouse, or was touching, smelling, or licking the face or anogenital region of the juvenile. Following this 1‐min trial, the stimulus mouse was removed and placed back into its holding cage for 10 min. The 1‐min social interactions followed by 10‐min breaks were then repeated for an additional four trials. However, on the last trial, that is, the “dishabituation” trial, the now familiar stimulus mouse was replaced by a novel juvenile mouse.

### 2.7 Operant paradigms

Operant paradigms were designed to determine the social motivation of the mice and have been previously validated for this purpose (Martin & Iceberg, 2015; Martin, Sample, Gregg, & Wood, 2014). For each stage of the operant paradigms (except shaping), the mice were tested for 20 consecutive days, 7 days a week.

#### 2.7.1 Shaping stage

During the shaping stage, the test mice were conditioned to press a lever in return for a social reward. The social reward took the form of 15 s of access through the wire grid to a social partner, which was a C57BL6 stimulus mouse. Each shaping session was 30 min long. After the mice were able to reward themselves by lever pressing 10 times for either two consecutive or three of five days, they advanced to the testing stage.

#### 2.7.2 Social motivation stage

The first testing stage of the operant paradigms was the social motivation stage. During testing, the mice were rewarded for lever presses using a progressive ratio schedule of reinforcement. The ratio schedule increased by a fixed rate of three every trial. Each daily trial continued until the test mouse ceased lever pressing for five consecutive minutes. Upon ending of the daily trail, the last reinforced ratio was recorded as the breakpoint.

#### 2.7.3 Valence comparison stage

The second stage of the operant paradigms was the valence comparison stage. Test mice were randomly assigned to either a left lever or right lever social reward group. The opposite lever for each group was associated with a food reward. The mice were given 6 days of discrimination training where they learned to associate one lever with a social reward and the other with a food reward. The social reward was the same as that used for the social motivation stage, a 15‐s interaction with a social partner. The food reward consisted of 15 s of access to sucrose/evaporated milk solution described above. After the 6th day of discrimination training, the mice were tested in 1‐hr daily sessions with both levers active. Each lever had a separate fixed rate of three for every trial.

### 2.8 Statistical analysis

All data were analyzed using IBM SPSS version 20.0 or later. Parametric statistical models including independent and paired samples *t* tests as well as repeated measures analysis of variance were used as appropriate to compare dependent measures across the levels of the independent variables.

## 3 RESULTS

### 3.1 Open‐field tests

**Figure 1:**
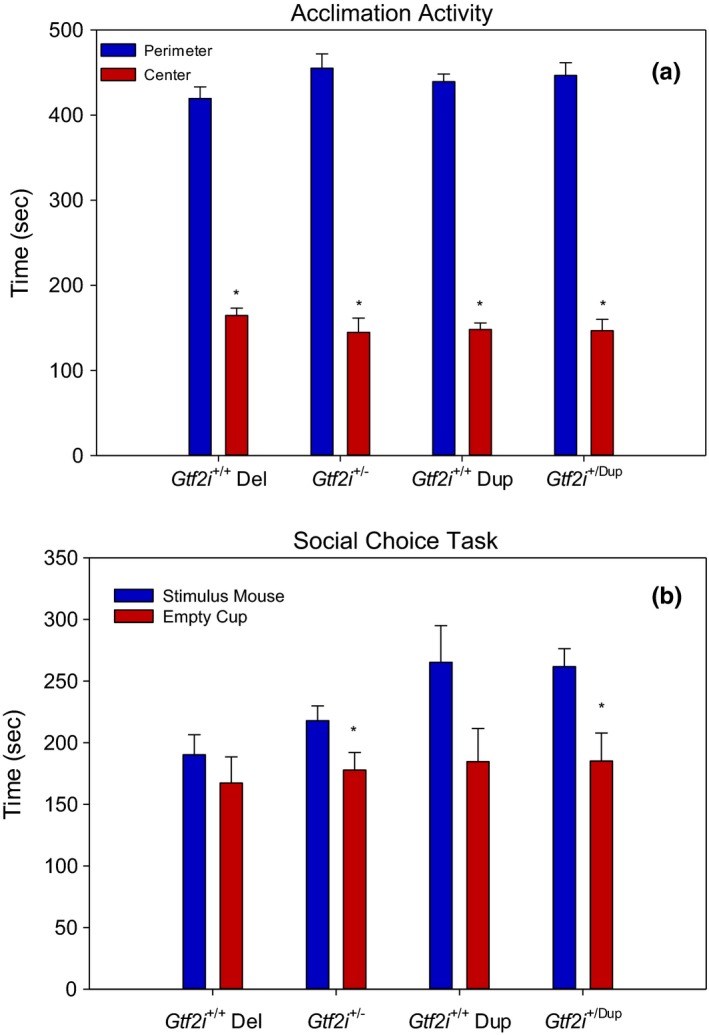
(a) Mean distance traveled and time spent in perimeter versus center of a novel open‐field environment. All four genotypes traveled similar distances and demonstrated a preference for the perimeter versus the center zones. (b) Mean time spent in stimulus mouse corner versus empty cup corner. The *Gtf2i* ^+/+^ mice did not show a corner preference, but the *Gtf2i* ^+/−^ mice and the *Gtf2i* ^+/dup^ mice spent significantly more time in the stimulus mouse corner than the empty cup corner. Asterisks indicate significance where *p* < .05

Comparisons were made between 15 *Gtf2i* ^*+/−*^ mice and 14 *Gtf2i* ^*+/+*^ siblings from Any‐maze tracking data obtained during a 10‐min trial in a novel open‐field arena. As shown in Figure 1a, the mean distance traveled during the 10‐min trials was similar between the two strains, indicating normal activity levels for the *Gtf2i* ^*+/−*^ mice. In addition, normal thigmotaxis was observed as paired samples *t* tests demonstrated that each strain spent significantly more time in the perimeter than the center of the arena (*Gtf2i* ^*+/+*^: *t*(13) = 12.06, *p* < .001; *Gtf2i* ^+/−^: *t*(14) = 9.31, *p* < .001). Furthermore, the time spent in each of these respective zones was very similar between the strains. During the social choice task, the *Gtf2i* ^*+/+*^ mice did not demonstrate the expected preference for the stimulus mouse over the empty cup; however, the *Gtf2i* ^+/−^ mice did spend significantly more time in the corner with the stimulus mouse than the empty cup corner (*t*(14) = 2.12, *p* = .05; see Figure 1b).

Comparisons were also made between 10 *Gtf2i* ^*+/dup*^ mice and 14 *Gtf2i* ^*+/+*^ siblings. The mean distance traveled in an open‐field arena was similar between the two strains, and paired samples *t* tests demonstrated that each strain spent significantly more time in the perimeter of the arena rather than the center (*Gtf2i* ^*+/+*^: *t* = 18.07, *df* = 13, *p* < .001; *Gtf2i* ^*+/dup*^: *t* = 10.64, *df* = 9, *p* < .001; see Figure 1c). The *Gtf2i* ^*+/dup*^ mice did not show the expected preference for the empty corner over the stimulus cup, instead they demonstrated the opposite (*t* = 2.49, *df* = 9, *p* = .034), while their wild‐type siblings showed no preference for either the empty cup or the stimulus mouse (see Figure 1d).

### 3.2 Habituation–dishabituation

**Figure 2:**
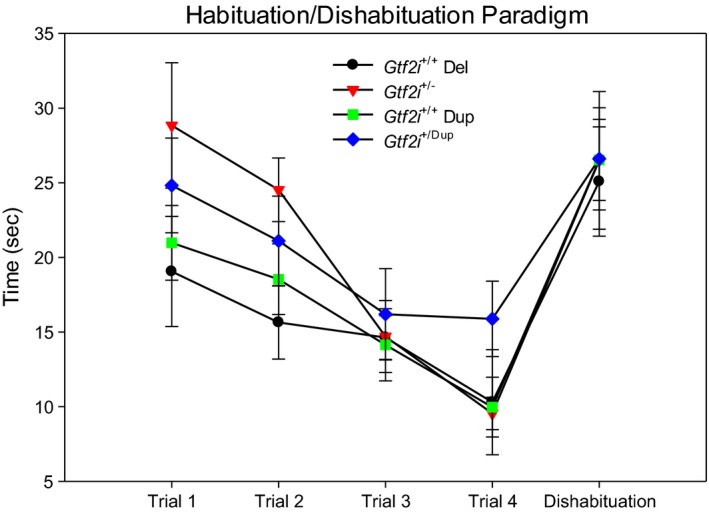
Mean time spent in each trial investigating a social stimulus partner. While the pattern was similar between mouse strains, the *Gtf2i* ^+/−^ mice demonstrated increased time spent in exploration of the social partner during trial 2 compared to *Gtf2i* ^+/+^ siblings. There was no significant difference in time spent with a social partner between the *Gtf2i* ^+/dup^ mice and their *Gtf2i* ^+/+^ siblings. For the dishabituation trial, all four mouse strains demonstrated the predicted rebound in social exploration time from the previous trial

Social recognition was also determined in eight *Gtf2i* ^+/−^ mice and eight wild‐type siblings as well as in 10 *Gtf2i* ^+/+^ mice and their 14 wild‐type siblings by measuring the habituation to a familiar social partner over a series of four 1‐min trials followed by a 5th 1‐min trial in which dishabituation to a novel partner was measured. As shown in Figure 2, all four strains of mice demonstrated a decline in social investigation across trials 1–4, but this decline was only significant for the *Gtf2i* ^+/−^ mice (*F*_3,21_ = 19.32, *p* < .001). Compared to *Gtf2i* ^+/+^ siblings, the *Gtf2i* ^+/−^ mice demonstrated a trend for increased exploration of the social partner on trial 1, which was significant by trial 2 (*t*(14) = 2.74, *p* = .016), and which disappeared by trials 3 and 4. No significant difference in investigation time was found between the *Gtf2i* ^*+/dup*^ mice and their wild‐type siblings. There was no difference in social exploration time on the dishabituation trial as all four mouse strains demonstrated a significant rebound from the previous trial (*Gtf2i* ^+/+^: *t*(7) = −2.96, *p* = .021; *Gtf2i* ^+/−^: *t*(7) = −3.63, *p* = .008; *Gtf2i* ^*+/+*^: *t* = −4.59, *df* = 15, *p* < .001; *Gtf2i* ^*+/dup*^: *t* = −2.23, *df* = 9, *p* = .053).

### 3.3 Social transmission of food preference

**Figure 3:**
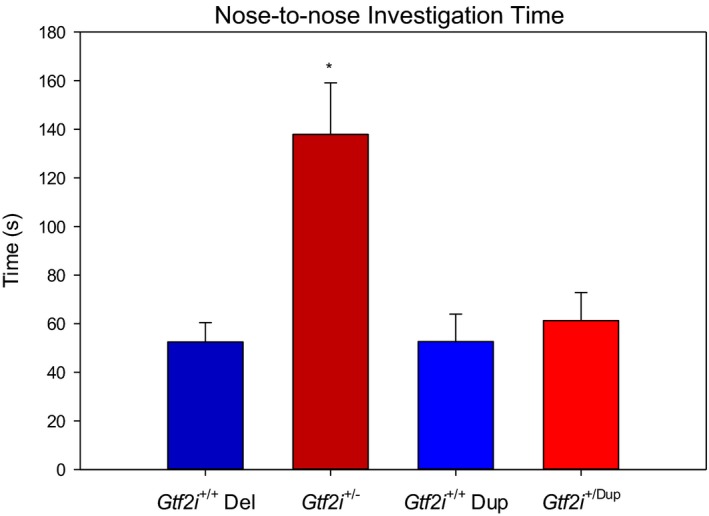
Mean time spent in nose‐to‐nose contact with demonstrator mouse during the STFP paradigm. *Gtf2i* ^+/−^ mice spent significantly more time in nose‐to‐nose contact than *Gtf2i* ^+/+^ siblings. There was no significant difference in nose‐to‐nose contact between the *Gtf2i* ^+/dup^ mice and their *Gtf2i* ^+/+^ siblings. Asterisks indicate significance where *p* < .05

*Gtf2i* ^*+/−*^ mice and *Gtf2i* ^*+/+*^ siblings demonstrated a similar success rate on the social transmission of food preference task with 67% of *Gtf2i* ^+/−^ mice (8 of 12) and 64% of *Gtf2i* ^+/+^ mice (7 of 11), showing preference for the food flavor of the demonstrator mouse. However, during the social encounter with the demonstrator mouse, the *Gtf2i* ^+/−^ mice exhibited significantly more nose‐to‐nose social encounters (*M* = 97 vs. 62; *t*(21) = −2.79, *p* = .011) and, similarly, spent significantly more time in nose‐to‐nose contact than their *Gtf2i* ^+/+^ siblings (*M* = 137.9 vs. 52.5 s; *t*(13.98) = −3.78, *p* = .002; see Figure 3). *Gtf2i* ^*+/dup*^ mice and *Gtf2i* ^*+/+*^ siblings also showed a preference for the food flavor found on the demonstrator mouse. However, no significant difference was found in the nose‐to‐nose contact time between these strains (Figure 3).

### 3.4 Olfactory probes

Normal olfactory ability was assessed using a buried food probe. The amount of time that each mouse took to find a buried Cocoa Puff (General Mills) was compared between genotypes. The buried food probe followed the social transmission of food preference task so that the cocoa scent was novel during STFP but a familiar scent for the buried food probe. There were no significant differences observed in the amount of time that it took each strain to find the buried Cocoa Puff suggesting normal olfactory ability for the *Gtf2i* ^+/−^ and *Gtf2i* ^*+/dup*^ mice.

**Figure 4:**
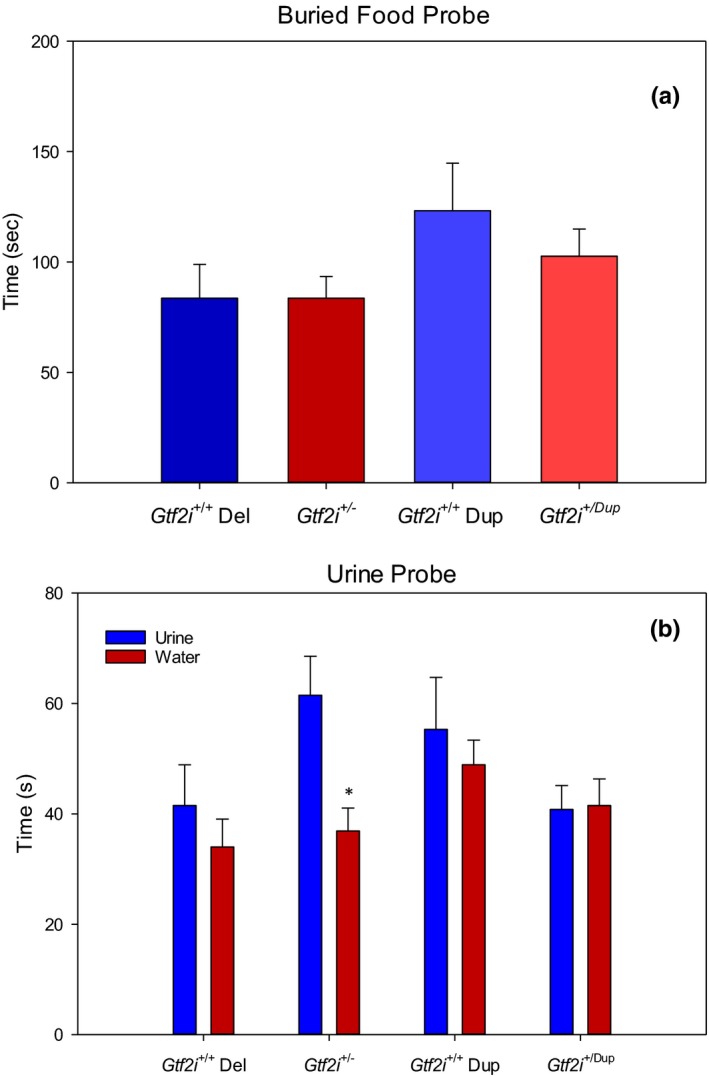
(a) Mean latency to find buried food. There were no significant differences between genotypes in the latency to find the buried Cocoa Puff. (b) Mean time spent in urine and water zones. *Gtf2i* ^+/−^ mice spent significantly more time in the urine zone versus the water zone, but there were no significant differences for the *Gtf2i* ^+/+^ mice. There was no significant difference in time spent in the urine zone versus the water zone between the *Gtf2i* ^+/dup^ mice and their *Gtf2i* ^+/+^ siblings. Asterisks indicate significance where *p* < .05

For the urine scent probe, the group of 15 *Gtf2i* ^+/−^ mice demonstrated a significant preference for the urine slide over the waterslide (*t*(14) = 4.31, *p* = .001), but there was no significant preference demonstrated by the group of 14 *Gtf2i* ^+/+^ mice. There was no significant difference in the time spent on investigating the urine and the waterslides for the *Gtf2i* ^*+/dup*^ mice or their *Gtf2i* ^*+/+*^ siblings. Figure 4b shows the mean time spent in the urine and water zones. Overall, the *Gtf2i* ^+/−^ mice spent more time in the urine zone (*M* = 61.5 s, *SD* = 27.3) than the *Gtf2i* ^+/+^ mice (*M* = 41.5, *SD* = 27.7); however, this difference only approached significance (*t*(27) = −1.96, *p* = .061).

### 3.5 Operant paradigms

#### 3.5.1 Social motivation paradigm

**Figure 5:**
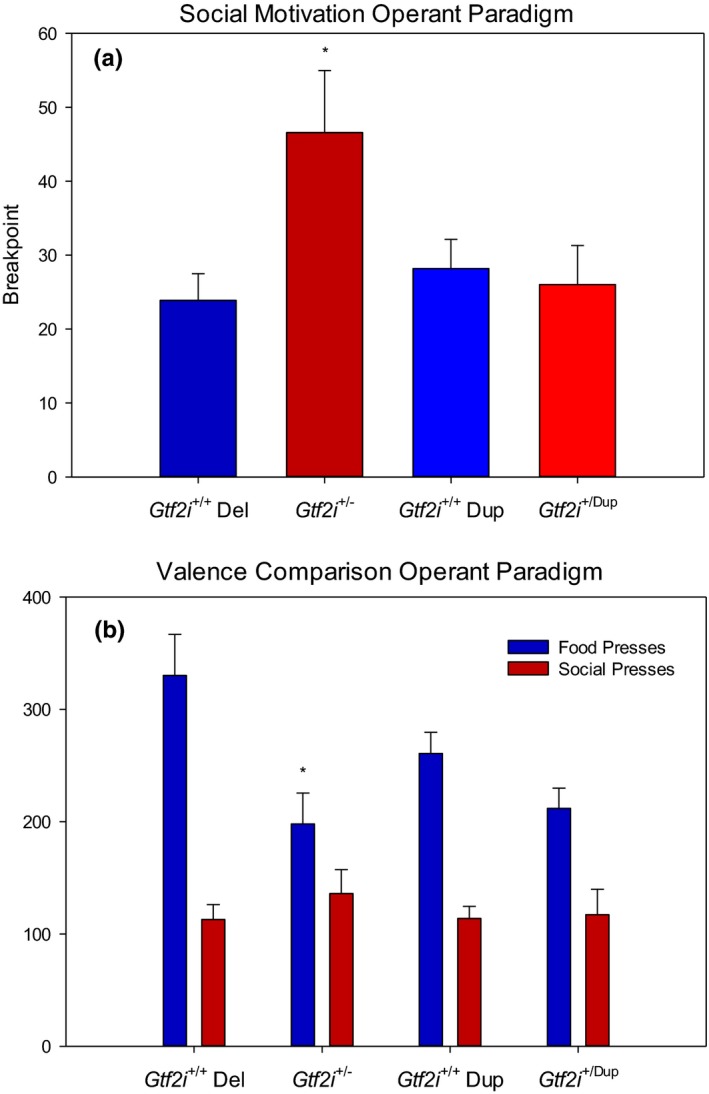
(a) Mean breakpoint (last reinforced ratio) achieved before the mice stopped lever pressing for 5 min. *Gtf2i* ^+/−^ mice demonstrated a significantly higher breakpoint than *Gtf2i* ^+/+^ siblings. There was no significant difference in breakpoint between the *Gtf2i* ^+/dup^ mice and their *Gtf2i* ^+/+^ siblings. (b) Mean number of presses for each type of reward. There was a significant preference for food over social rewards for both genotypes. However, *Gtf2i* ^+/−^ mice demonstrated significantly fewer food presses and made a significantly higher percentage of presses for social rewards than *Gtf2i* ^+/+^ siblings. There was no significant difference in percentage of presses for food and social rewards between the *Gtf2i* ^+/dup^ mice and their *Gtf2i* ^+/+^ siblings. Asterisks indicate significance where *p* < .05

Both genotypes learned to associate lever pressing with a social reward during the shaping phase of the social motivation paradigm. There was no significant difference in the number of days required to reach criterion for advancement to the testing stage between *Gtf2i* ^+/−^ (*M* = 7.1, *SD* = 3.8) and *Gtf2i* ^+/+^ siblings (*M* = 8.3, *SD* = 4.1) or between *Gtf2i* ^*+/dup*^ (*M* = 12.3, *SD* = 11.8) and *Gtf2i* ^+/+^ siblings (*M* = 9.2, *SD* = 5.0). Results from the progressive ratio stage revealed a significantly higher mean breakpoint for the group of 14 *Gtf2i* ^+/−^ mice (*M* = 46.6, *SD* = 31.4) compared to the group of 14 *Gtf2i* ^+/+^ siblings (*M* = 23.9, *SD* = 13.6; *t*(17.73 = −2.49, *p* = .023); See Figure 5a). There was no significant difference in mean breakpoint between 10 *Gtf2i* ^*+/dup*^ mice (*M* = 26.0, *SD* = 15.8) and their 14 *Gtf2i* ^*+/+*^ siblings (*M* = 28.2, *SD* = 17.2).

#### 3.5.2 Valence comparison paradigm

All four genotypes successfully learned to discriminate between lever and reward contingencies for food and social rewards. As shown in Figure 5b, the mean number of lever presses for the food reward was 330 (*SD* = 137.2) for the 15 *Gtf2i* ^*+/+*^ mice, 198 (*SD* = 102.9) for their 14 *Gtf2i* ^*+/−*^ siblings, 211.8 (*SD* = 54.3) for the 10 *Gtf2i* ^*+/dup*^ mice, and 260.7 (*SD* = 82.4) for their 14 *Gtf2i* ^*+/+*^ siblings. Figure 5b also shows the mean number of lever presses for the social reward was 113 (*SD* = 49.5) for the *Gtf2i* ^*+/+*^ mice, 136 (*SD* = 79.4) for the *Gtf2i* ^*+/−*^ mice, 117 (*SD* = 67.9) for the *Gtf2i* ^*+/dup*^ mice, and 113.8 (*SD* = 46.9) for their *Gtf2i* ^*+/+*^ siblings. Paired samples *t* tests revealed significant preferences for food over social rewards for all four genotypes. However, *Gtf2i* ^*+/−*^ mice demonstrated significantly fewer food presses (*t* = 2.89, *df *= 26, *p* = .008) and made a significantly higher percentage of presses for social rewards than *Gtf2i* ^*+/+*^ siblings (*t* = −2.86, *df *= 26, *p* = .008). There was no difference in percentage of presses for food and social rewards between the *Gtf2i* ^*+/dup*^ mice and their *Gtf2i* ^*+/+*^ siblings.

## 4 DISCUSSION

The behavioral results from multiple testing paradigms consistently demonstrated increased social behavior of *Gtf2i* ^+/−^ mice compared to *Gtf2i* ^+/+^ siblings. *Gtf2i* ^+/−^ mice demonstrated a significant preference for a stimulus mouse over an empty cup in the social choice task, spent significantly more time in nose‐to‐nose contact with the demonstrator mouse in the social transmission of food preference task, spent significantly more time investigating the urine scent versus water in the urine scent probe, and demonstrated heightened social investigation time in early trials of the habituation–dishabituation paradigm. All of these tasks involve tracking behaviors as the test mice move freely in an open‐field or home‐cage environment. However, our operant paradigms involve a more rigorous investigation of social motivation through their requirement of greater effort from the test mouse in its attempt to gain a social reward. *Gtf2i* ^+/−^ mice demonstrated significantly more lever presses for a social reward in the social motivation operant paradigm and made a significantly higher percentage of lever presses for a social reward in the valence comparison operant paradigm compared to *Gtf2i* ^+/+^ siblings. These operant paradigms stand apart from the other behavioral assessments in their level of construct validity for the measurement of social motivation (Martin et al., 2014). Furthermore, these paradigms offer face validity to a growing number of testing paradigms used to measure social motivation in humans including those referenced earlier for the study of ASD.

The comparisons of the *Gtf2i* ^*+/dup*^ mice to *Gtf2i* ^*+/+*^ siblings did not provide support for decreased social behavior in mice carrying the *Gtf2i* duplication. It was found that the *Gtf2i* ^*+/dup*^ mice demonstrated a preference for the stimulus mouse over the empty cup in an open‐field arena that their *Gtf2i* ^*+/+*^ siblings did not, but this was counter to our hypothesis. *Gtf2i* ^+/dup^ mice and their *Gtf2i* ^*+/+*^ siblings displayed expected patterns of habituation/dishabituation to a social partner and demonstrated social transmission of food preference, further contradicting the hypothesis of a hyposocial phenotype. Additionally, neither strain showed a significant preference for the urine zone over the water zone during the olfactory probes. *Gtf2i* ^+/dup^ mice and their *Gtf2i* ^*+/+*^ siblings demonstrated similar amounts of lever presses for a social reward in the social motivation operant paradigm and demonstrated a similar percentage of social presses and food presses in the valence comparison operant paradigm, preferring a food reward over a social reward. Overall, the results from the various assays do not support the hypothesis of a hyposocial phenotype in *Gtf2i* ^*+/dup*^ mice, although this does not eliminate the potential for other ASD‐like traits in these mice such as heightened separation anxiety as has been previously reported (Mervis et al., 2012).

While social behavior is a complex construct that can be influenced by many underlying factors, nonsocial behavioral measures were remarkably consistent across sibling comparisons. We did not find any differences in locomotor activity or thigmotaxis in an open‐field environment suggesting normal levels of activity and anxiety. We also found evidence in support of normal olfactory ability in the buried food probe. All four groups also learned to lever press for a social reward over a similar number of training sessions suggesting intact learning ability. Finally, the decreased number of lever presses for a food reward observed in the valence comparison paradigm countered any potential hypothesis of a general increase in motivated behavior for the *Gtf2i* ^+/−^ mice. Together, these results suggest that the observed differences in social behavior between *Gtf2i* ^+/−^ and *Gtf2i* ^+/+^ mice are more directly associated with changes in GTF2I expression. Previous research supports a linear relationship between *Gtf2i* copy number and GTF2I expression across *Gtf2i* ^+/−^, *Gtf2i* ^+/+^, and *Gtf2i* ^*+/dup*^ mice (Mervis et al., 2012). Our results suggest that GTF2I expression does not have a linear relationship with social behavior.

The way in which GTF2I ultimately influences social behavior is likely complex. However, GTF2I is widely expressed in the brain including the ventral tegmental area (VTA), nucleus accumbens (NAc), and amygdala (Science, 2004). The connection of the VTA to the NAc is well known for its role in the experience of reward, and more recently, elevated activity of dopaminergic VTA neurons projecting to the NAc has been shown to predict social interaction in mice (Gunaydin et al., 2014). Furthermore, reduced dopaminergic activity in the VTA in a model of *Shank3* insufficiency was shown to impair social preference, while optogenetic stimulation of these same neurons resulted in enhanced social preference behavior (Bariselli et al., 2016). Collectively, these results suggest a potential target for the influence of GTF2I on social behavior in the VTA to NAc circuitry. GTF2I influences on social behavior may also be mediated through the amygdala. Recent research on *GTF2I* polymorphisms has demonstrated a connection between common variations and reduced social anxiety (Crespi & Hurd, 2014). This reduced social anxiety has further been linked to reduced threat‐related amygdala reactivity and the personality dimension of warmth in female participants (Swartz et al., 2017). The authors even propose a potential molecular target for GTF2I in the amygdala, the serotonin receptor 3A (*HTR3A*), a known transcriptional target of GTF2I regulation (Segura‐Puimedon, Borralleras, Perez‐Jurado, & Campuzano, 2013).

The consistent heightened social behavior observed in *Gtf2i* ^+/−^ mice solidifies the importance of *GTF2I* in the regulation of social motivation, and yet, the consistency of these findings stands in stark contrast to the lack of differences observed in *Gtf2i* ^+/dup^ mice. Future studies should focus on the molecular mechanisms of GTF2I interaction in an effort to determine why duplication of the gene did not have any major impacts on social behavior. Additional attempts at observing a dosage effect of *Gtf2i* in mice should also be explored. Mice with 200% Gtf2i expression may exhibit a hyposocial phenotype that was not seen in the *Gtf2i* ^+/dup^ mice in this study with presumed 150% expression (Mervis et al., 2012). Further research may employ inducible techniques to examine whether or not changes in social behavior can still be observed with the reduction in *Gtf2i* in adult mice and whether the complete elimination of *Gtf2i* is lethal in mature animals as has been observed in embryonic mice carrying a homozygous deletion of *Gtf2i* (Sakurai et al., 2011).
